# On Meteorical, Stony, and Metalline Substances

**Published:** 1804-04-01

**Authors:** 

**Affiliations:** Berlin


### On Meteorical, Stony, and Metalline Substances; *by Mr. Klaproth, of Berlin.*

A VARIETY of facts has now rendered it indisputable, that stony and metalline substances have fallen on the earth at different times, however miraculous such a phenomenon may appear, and however it may differ from what is observed as the common order of Nature. The physico-chemical properties likewise which these substances possess, apparently declare their identity, and are capable of throwing some light on their origin and mode of formation. Any contribution, therefore, towards explaining so remarkable a subject must be acceptable to the philosophical naturalist. Mr. Klaproth, whose name ranks high amongst the modern chemists, having obtained a sufficient quantity of those substances, which have fallen on on different parts of our globe, took an opportunity of submitting them to a chemical analysis, the results of which he communicated to the Royal Academy at Berlin, and are as follow:

I. *Analysis of the Meteorical Stones from Siena.*

The account of the curious phenomenon by which these stones were thrown on the earth is sufficiently known. The accident happened in June 1794, near Siena, in Italy, where, about seven o'clock in the evening, a small dark cloud was perceived in the sky, which otherwise remained clear. Soon afterwards a violent explosion took place, attended with lightening, which resembled the discharge of a battery. At each explosion a considerable motion was seen in the nebula which surrounded the cloud, and a great quantity of stones fell down, most of which were small, but some were found of seven pounds weight. The force of the fall was so great that it occasioned a hissing noise in the atmosphere, and the greatest part of the stones was found buried several feet deep in the ground, which had become soft by the preceding rains.

Mr. Klaproth received several specimens of those stones; they were all covered with a thin crust of a grey blackish colour, without gloss, and of a rough and uneven surface. When these stones are broken, they show a very dissimilar texture; the chief substance, however, is ash coloured, and has the appearance of hardened argilla, but not the least smell of it. The particles that are discovered in it, consist of four different substances, the most remarkable of which is iron in a metallic state, found particularly near the crust in form of small eliptical grains. They may be extended by means of a hammer, and the file stroke appears white and glossy. The second substance is martial pyrites, which is distributed in small particles and veins, and has a reddish-yellow colour. The third substance is mixed with the whole in a greater proportion, and consists of small bodies of various sizes, mostly flat and angular. They have a considerble hardness, a black, grey, or brownish colour, a conchoid fracture, and a small degree of lustre. Besides these small globular bodies are perceived, of a yellowish colour and a vitreous lustre; much resembling quartz without having its hardness. The whole stone has not the least similarity with any of the known fossils, or rocks. The specific weight is between 3,340 and 3,400.

1. Two

1. Two hundred grains of the meteorical stone from Siena having been grossly pulverised in a glass mortar, the globules of iron were separated by means of the magnet, and weighed about six grains and a half, which were infused in muriatic acid, and the solution promoted by a gentle heat; during this process sulphurated hydrogen gas was disengaged, which arose from the small particles of martial pyrites that adhered to the iron grains. The clear solution had the green colour of emerald. On being precipitated, and afterwards supersaturated with caustic ammonia, the liquor became light blue. This liquor, after being separated from the precipitated oxyd of iron, was evaporated to dryness, and the remaining greenish salt ignited in a platina crucible, by which it was changed into a soft micacious powder, and gave a green solution with nitric acid. On adding to it carbonat of kali, three grains and a quarter of oxyd of nickel were precipitated; of which, after ignition, remained 1,60 gr. of a greyish-green oxyd of nickel, equal to 1,20 of metallic nickel. The oxyd of iron, after being ignited in a close crucible, appeared blackish, and weighed six grains and a quarter, which is equal to about four grains and a half of metallic iron.

2. The remaining 193½ grains of the stone, after being finely pulverised, were extracted by repeatedly infusing and digesting them with muriatic acid, whereby a disengagement of sulphurated hydrogen gas was occasioned. The colour of the liquor was straw-yellow. The greyish-white residuum being mixed with three times as much of caustic natron, and ignited, was softened with water, then supersaturated with muriatic acid, and afterwards evaporated to dryness. It was now again mixed with water, and the insoluble part separated by means of the filtrum, edulcorated, dried, and ignited. It weighed eighty-eight grains, and consisted of siliceous earth.

3. A precipitation was effected in the muriated solution when boiling, by means of carbonat of kali, which appeared dark green. Caustic ley, with which it was boiled, had no remarkable effect on it. After the precipitate had been sufficiently edulcorated, it was dissolved in sulphuric acid, and the solution evaporated to dryness; the salt thus obtained was strongly ignited, by which means it became pale red, and dusty. It was boiled with distilled water and filtrated; the residuum being edulcorated and dried was mixed with oil and made red hot in a close vessel, by which which means fifty grains of a black oxydulated iron were obtained.

4. The remaining sulphurated liquor, when boiling, was decomposed by carbonat of kali, by which means a precipitate, consisting of magnesia, was obtained; it weighed forty-five grains and a half. The earth was soft and ash-coloured. On dissolving it in sulphuric acid, a black precipitate fell down, consisting of oxyd of manganese, which after ignition weighed half a grain. The liquor being freed from this oxyd became quite clear, and deposited crystals of sulphat of magnesia. The proportions of the constituent parts of the stone from Siena are in 100 parts as follows,

| | |
|---|---|
| Perfectly metallic iron - - - | 2,25 |
| Metallic nickel - - - - - - | 0,60 |
| Black oxyd of iron - - - | 25, |
| Magnesia - - - - | 22,50 |
| Siliceous earth - - - | 44, |
| Oxyd of manganese - - | 0,25 |
| Loss, including the sulphur and oxyd of nickel - | 5,40 |
| | 100. |

The quantity of the oxyd of nickel, as well as of the sulphur was too small to be accurately estimated: The presence of sulphur was decidedly indicated by the disengagement of the sulphurated hydrogen gas.—A piece of meteorical stone being put into an earthen crucible, was exposed to the fire of a porcelain furnace; where it touched the crucible it was found to have melted with it, but in the middle it was changed into a spongy dross of a moderate metallic lustre.

### *Analysis of a Meteorical Stone from Aichstaedt in Germany.*

The account of this substance is related by Baron Hompesch as follows. "In winter, when the earth was covered one foot deep with snow, a labourer who worked in the fields saw this stone fall down, immediately after a preceding thunderstroke. He instantly ran to the place where it had fallen, and endeavoured to take it up, but it was so hot, that he could not touch it, and was obliged to let it cool. The stone was about half a foot in diameter, and covered with a black crust of iron. It greatly resembles the stone of Siena with respect to its external crust as well as the internal structure, but the particles of martial pyrites rites are changed into a brown oxyd of iron. It was examined in a similar manner with the former, and the constituent parts were obtained in the following proportion.

| | |
|---|---|
| Perfectly metallic iron - | .19, |
| Metallic nickel - - | 1,50 |
| Brown oxyd of iron - | 16,50 |
| Magnesia - - - | 21,50 |
| Siliceous earth - - | 37, |
| Loss, including the sulphur and oxyd of nickel - | 4,50 |
| | 100. |

No trace of sulphat of kali, or natron could be discovered in this substance.

2. *Analysis of the Meteorical Iron from Sclavonia.*

This remarkable mass of metallic iron is preserved in the Imperial Museum at Vienna; it has the form of an irregular triangle, and weighs seventy-one pounds. The external surface is full of deep impressions, but the inside appears solid, like hammered iron, of a zinc- white colour and a considerable metallic lustre. The account of the phenomenon by which this mass was thrown down, is the following. On May 26th, 1751, about six o'clock in the evening, a fiery globe was perceived in the atmosphere near Itraskina, in the Bishoprick of Agram in Sclavonia, which, with a violent explosion, separated into two parts, and fell down in form of two twisted chains with a great noise. The larger piece, which first came down, had penetrated into the earth with such force, that it caused a kind of earthquake in the adjoining ground. It had occasioned a chasm three fathoms deep and one yard broad. The second piece weighed sixteen pounds, and had fallen in a meadow two thousand paces distant from the other, where it had occasioned a similar chasm. The Bishop of Agram, to whom the larger piece had been presented, sent it to the Imperial Museum of Vienna. Mr. Klaproth having obtained a piece from that mass, submitted it to a chemical analysis.

1. A hundred grains of this iron being infused in muriatic acid were put in digestion. The solution proceeded slowly without the least disengagement of sulphurated hydrogen gas; it had a beautiful emerald-green colour. In order to oxydate still more the dissolved iron, some nitric acid was gradually added to the solution till no

 effervescence effervescence ensued, by which means the green colour was changed into red-brown, and the iron perfectly dissolved.

2. The solution was supersaturated with ammonia, and the oxyd of iron, thus precipitated, was digested with weak caustic ley, edulcorated, dried, and ignited for half an hour. In this state of red-brown oxyd of iron it weighed one hundred and forty-two grains, which comes near to ninety-six and a half of metallic iron.

3. The ammoniacal liquor appeared light-blue, and being sufficiently evaporated, the greenish salt mass that remained was gently ignited, till no fumes were any longer disengaged. The residuum being dissolved in nitric acid formed a greenish solution, in which a precipitation was effected by carbonat of kali, and the oxyd of nickel thus obtained was ignited. It weighed one three-quarter grain, which indicates three grains and a half of metallic nickel. The proportion of the constituents are, in one hundred parts,

| | |
|---|---|
| Metallic iron — — — — | 96,50 |
| Metallic nickel — — — — | 3,50 |
| | 100. |

4. *Analysis of the Meteorical Iron from Siberia.*

It is very probable that the mass of iron which was first discovered by the celebrated Pallas has the same origin with the former. It is said to weigh one thousand six hundred pounds. It is covered with a rough crust of iron, but internally consists of metallic iron, in whose cellular interstices a yellowish fossil, similar to olivin or crysolithes, is disseminated. In order to know whether this iron equally contained nickel, it was treated in the same manner as the former. After the precipitation of the oxyd of iron by caustic ammonia the remaining liquor appeared light-blue, and left, after evaporation, a greenish salt mass, from which, after the evaporation of the muriat of ammonia, the nickel was separated. The proportion of both metals, in one hundred parts, was

| | |
|---|---|
| Metallic iron — — | 98,50 |
| Metallic nickel — — | 1,50 |
| | 100. |

Of the yellowish substance belonging to the Siberian iron, one hundred grains were infused with three hundred grains of concentrated sulphuric acid and the same quantity tity of water, and after sufficient digestion distilled, and the liquor was cohobated. The residuum was mixed with boiling water, to which some sulphuric acid had been added, and afterwards filtrated. Siliceous earth remained, which weighed after ignition forty-one grains. The sulphuric liquor was evaporated to dryness, and the residuum strongly ignited, by which it became pale red and dusty. Being infused with water, and filtrated, a red oxyd of iron remained, which after being edulcorated, dried, and mixed with oil, gave, after ignition, eighteen grains and a half of black oxyd of iron. The liquor, which was free from iron, deposited crystals of sulphat of magnesia, and the magnesia obtained from it weighed, after ignition, thirty-eight grains and a half; one hundred parts, therefore, contained,

| | |
|---|---|
| Siliceous earth — — | 41, |
| Magnesia — — — | 38,50 |
| Oxyd of iron in an attractable state | 18,50 |
| | 98. |

The author comparing in the second section of his Treatise his analysis with those made by other chemists on these meteorical substances, states, that except in the quantitative proportions, they perfectly agree with his examinations; a circumstance which seems to prove the common origin of all those substances. In the third section he relates several instances of the fall of meteorical substances, which he found recorded, as an addition to those mentioned by Mr. Chladni.

In the fourth section the author relates the opinions of several naturalists on the origin of meteorical substances, viz. La Place's, Chladni's, &c.

Mr. Klaproth concludes his paper with examining the native iron from Karnsdorf in Saxony, in order to find out the difference between this and the meteorical iron. According to the results of his analysis, the native iron of Karnsdorf contains, in one hundred parts,

| | |
|---|---|
| Iron — — — | 92,50 |
| Lead — — — | 6, |
| Copper — — — | 1,50 |
| | 100. |

From these experiments it results, that the presence or absence of nickel may serve as a criterion for discovering any native iron from such iron as is of a meteorical origin

